# Pro-Inflammatory Derangement of the Immuno-Interactome in Heart Failure

**DOI:** 10.3389/fimmu.2022.817514

**Published:** 2022-03-15

**Authors:** Pavanish Kumar, Amanda Lim, Su Li Poh, Sharifah Nur Hazirah, Camillus Jian Hui Chua, Nursyuhadah Binte Sutamam, Thaschawee Arkachaisri, Joo Guan Yeo, Theo Kofidis, Vitaly Sorokin, Carolyn S. P. Lam, Arthur Mark Richards, Salvatore Albani

**Affiliations:** ^1^ Translational Immunology Institute, SingHealth Duke-NUS Academic Medical Centre, Singapore, Singapore; ^2^ KK Research Centre, KK Women’s and Children’s Hospital, Singapore, Singapore; ^3^ Paediatrics Academic Clinical Programme, SingHealth Duke-NUS Academic Medical Centre, Singapore, Singapore; ^4^ Rheumatology and Immunology Service, KK Women’s and Children’s Hospital, Singapore, Singapore; ^5^ National University Heart Centre, Singapore, Singapore; ^6^ The National University Health System (NUHS) Cardiovascular Research Institute, Singapore, Singapore; ^7^ Duke-NUS Medical School, Cardiovascular Academic Clinical Program, Singapore, Singapore; ^8^ National Heart Centre, Singapore, Singapore

**Keywords:** heart failure, cardiovascular disease, systems immunology, CyTOF, network biology, inflammation, immunity

## Abstract

Chronic heart failure (HF) is a syndrome of heterogeneous etiology associated with multiple co-morbidities. Inflammation is increasingly recognized as a key contributor to the pathophysiology of HF. Heterogeneity and lack of data on the immune mechanism(s) contributing to HF may partially underlie the failure of clinical trials targeting inflammatory mediators. We studied the Immunome in HF cohort using mass cytometry and used data-driven systems immunology approach to discover and characterize modulated immune cell subsets from peripheral blood. We showed cytotoxic and inflammatory innate lymphoid and myeloid cells were expanded in HF patients compared to healthy controls. Network analysis showed highly modular and centralized immune cell architecture in healthy control immune cell network. In contrast, the HF immune cell network showed greater inter-cellular communication and less modular structure. Furthermore, we found, as an immune mechanism specific to HF with preserved ejection fraction (HFpEF), an increase in inflammatory MAIT and CD4 T cell subsets.

## Introduction

Chronic HF is a clinical syndrome characterized by typical symptoms reflecting raised intracardiac pressures and/or insufficient cardiac output to meet systemic requirements at rest or on exertion (1, 2). The global prevalence of HF is increasing and among adverse cardiovascular events, acute HF is a leading cause of hospitalization (3). Left Ventricular (LV) Ejection Fraction (EF) has emerged as a useful clinical marker for categorizing HF patients. LVEF shows a bimodal distribution among patients with HF (4). The European Society of Cardiology (ESC) guideline (5) categorizes HF patients into three categories, HF with reduced EF (HFrEF; EF ≤ 40%), HF with preserved EF (HFpEF ≥ 50%), and HF with mildly reduced EF (HFmrEF; EF 41-49%) EF. Although HFpEF and HFrEF show similar bedside clinical phenotypes, the underlying pathogenesis is distinct (4, 6). Multiple comorbidities and Disease heterogeneity associated with HF complicate the diagnosis and treatment of the disease. Currently, guideline mandated, evidence-based therapies in HF are characterized by a paucity of proven biomarkers for HF patient’s stratification for effective and targeted treatment selection. Treatments of proven benefit in HFrEF do not improve survival in HFpEF (7). Moreover, the number of patients with HFpEF is rising. Lack of treatment options, heterogeneity in clinical presentation, and an increased prevalence rate of HFpEF call for a better understanding of the molecular pathology specific to HFpEF (6). In this context, inflammation plays an important role as a contributory pathogenic mechanism in HF. Elevated serum pro-inflammatory cytokine levels in HF patients were first reported in 1991 (8) and in the last 30 years, many more studies have recognized the role of inflammation in HF (9, 10). Local and systemic inflammation have both been associated with HF (11–14). Multi-system (including renin-angiotensin-aldosterone and sympatho-adrenal systems) neurohormonal activation and hemodynamic overload contribute to persistent myocardial and systemic inflammation in HF (15) suggesting a regulatory link between the immune and circulatory systems. Preclinical data (16, 17) have demonstrated TNF-α mediated left ventricular dysfunction and remodeling and cytokine-induced impaired cardiac myocyte contractile function. Subsequently, several clinical trials motivated by results from clinical observation and experimental studies, targeted cytokines to treat HF (18–20). However, these failed to demonstrate any benefit from anti-cytokine therapy. In contrast, in the recent CANTOS trial an anti-IL-1β monoclonal antibody (canakinumab) reduced HF-related hospitalizations and mortality in patients with previous myocardial infarction (21), but the system-wide effects and mechanisms of action remain undefined.

Altogether, the unmet medical needs and the knowledge gaps mandate further research and in-depth understanding of immune mechanisms associated with HF in humans.

The current report demonstrates evolution from studying individual immune-related variables to a more comprehensive approach to the systemic Immunome. We employed EPIC (22), a high dimensionality platform based on Cytometry by Time Of Flight (CyTOF) to study the immunome of HF patients, categorized in HFpEF and HFrEF, as well as matched healthy controls.

## Materials And Methods

### Human Subjects

Patients were recruited within the Singapore ATTRaCT HF consortium with participation by all major public hospitals in Singapore. Patients who underwent cardiovascular disease related surgical procedure and who had clinical history of HF were recruited for the study. The clinical diagnosis of heart failure was established based on European Society of Cardiology (ESC) criteria. All patients underwent comprehensive Doppler echocardiography, with left ventricular ejection fraction determined by Simpson’s biplane method and assessment of left ventricular diastolic dysfunction using standardized criteria (23). Healthy subjects were recruited at KK hospital Singapore. Peripheral venous blood samples were collected for the study. The study subjects comprised 34 HF patients and 18 healthy controls. The study protocol was reviewed and approved by NUHS, NHCS and KKH Central Institutional Review Board (CIRB). Informed consent was obtained according to the CIRB requirements. Full clinical and demographic information of the patients and samples used for various experiments is summarized in **Table S1**. Summary of sample demographics is shown in **Table 1**.

**Table 1:** Human subject demographics summary.

	HC (n=18)	HF (n=32)	HFpEF (n=14)	HFrEF (n=14)
**Age (years) median (sd)**	63.5 (10.67)	58.5 (10.54)	59 (11.73)	56.5 (9.88)
**Gender (F/M)**	6/12	3/32	1/13	0/14
**Left ventricular ejection fraction median ( sd)**	NA	49 (13.28)	56.5 (5.72)	40 (11.39)

NA, Not Available.

### Peripheral Blood Mononuclear Cells (PBMCs) Preparation

Peripheral blood samples were collected in ethylene diamine tetra acetic acid tubes (EDTA). PBMCs were isolated from blood samples using Ficoll-Paque (GE Healthcare) gradient centrifugation within 6 hrs from recruitment and were stored in liquid nitrogen until further use.

### CyTOF

Two panels of antibodies were designed and used for comprehensive immunome analysis: panel 1 antibodies focused on T cells and NK cells, while panel 2 was focused on B cells (**Tables S2**, **S3**). To reduce the technical variability and simultaneous sample processing, a combination of three anti-CD45 antibody barcodes were used, as previously described (18). The antibodies were either conjugated in-house according to the manufacturer’s instructions (Fluidigm) or purchased pre-conjugated directly from the supplier (Fluidigm). PBMCs were thawed and rested overnight in complete RPMI medium supplemented with 10% FBS, 1% penicillin/streptomycin/glutamine, and 10 mM Hepes at 37°C. The cells were then stimulated for 6 h with 150 ng/mL phorbol myristate acetate (PMA) and 100 ng/mL ionomycin (Sigma) and exposed to 3 μg/mL Brefeldin A (eBiosience) and 2 μM monesin (BioLegend) during the final 4 h of the incubation. Next, the cells were stained with cisplatin (Fluidigm) to identify live/dead cells and incubated with metal-conjugated surface-membrane antibodies. The cells were then fixed in 1.6% paraformaldehyde and permeabilized in 100% methanol to permit staining with intracellular metal-conjugated antibodies. Finally, the cells were labeled with an iridium-containing DNA intercalator before analysis on a CyTOF-II mass cytometer (Fluidigm).

The signal was bead normalized using EQ Four Element Calibration Beads (EQ Beads, 201078, Fluidigm), according to manufacturer’s instructions. The generated.fcs files were filtered for live/dead cells and DNA and then manually de-barcoded using FlowJO software.

### Data and Statistical Analysis

The de-barcoded files were analyzed in R statistical programming software (Version R-3.4). Data preprocessing and batch normalization were performed as described by Yeo, et al. (22). Briefly, data was normalized with cofactor of 5 for all analyses. Batch-wise scale normalization was performed to minimize any batch effect. For t-distributed Stochastic Neighbor Embedding (t-SNE) dimensionality reduction, each sample was down-sampled to 10,000 cells. The resulting cells were clustered into nodes using k-means clustering. Elbow method was used to determine the number of clusters. The Wilcoxon rank-sum test (coin R package) was used for statistical comparisons between HF and healthy controls. A p<0.05 was considered statistically significant.

### Network Analysis

The frequency of nodes (Immune cell subsets) for both panel1 and panel2 antibody staining data for each human subject was calculated. Proportions of T cells and NK cells nodes from Panel1 and B and mono/DC from Panel 2 were combined into a single matrix. Pairwise correlations between proportions of nodes were calculated for healthy control and HF samples. To construct the network, nodes were connected at an absolute threshold correlation coefficient >0.6. The network was visualized and analyzed using the igraph R package.

## Results

### The Architecture of the Systemic Immunome in Chronic HF Disease Is Deranged

We employed an *ad hoc* adaptation of our CyTOF-based EPIC platform (22). Peripheral blood mononuclear cells (PBMC) from patients with a history of HF and age-matched healthy controls (HC) were analyzed using 2 panels of antibodies. Panel 1(P1) was focused on T and NK cells, and Panel 2 (P2) was upon B and myeloid cells (**Tables S2**, **S3**). Each Panel comprised 36 markers with 19 markers in common to both P1 and P2. In total 53 unique surface and intracellular markers were evaluated. Markers were chosen to study all major lineage immune cells and key pro-and anti-inflammatory cytokines and various chemokines/cytokine receptor that also helps in identification of major lineage subsets and their trafficking potential. A summary of marker functional category is described in **Table 2**.

**Table 2:** Antibody marker functional group.

Functional group	Antibody markers
**T cell Markers**	CD4,CD8,CD3,CD28,CD 69,CD154,CD45 RA,CD45RO,CD27,CCR7,CD62L,Va7.2,CD161
**B cell Markers**	CD19,CD38,IgD,IgM,CD24,CD20
**Monocyte Markers**	HLA-DR,CD14,CD16,TLR4,CD11c,CD11b
**T Reg cell markers**	CD25,FOXP3,CD233,PD1
**NK cell Marker**	CD56
**Chemokine/cytokine trafficking receptors**	CCR6 , CCR4, CXCR5, CD103, CD31, cMet, AGTR2, CXCR3,CCR2
**Pro-inflammatory chemokine/cytokines**	TNF-α, IFN-γ, TGF-β, HMGB1, MIP1-β, IL-6, IL-8, IL-1β, IL-21, IL17A, IL22, Granzyme B,
**Anti-inflammatory chemokine/ cytokines**	IL-10, IL-4

**Figure 1:**
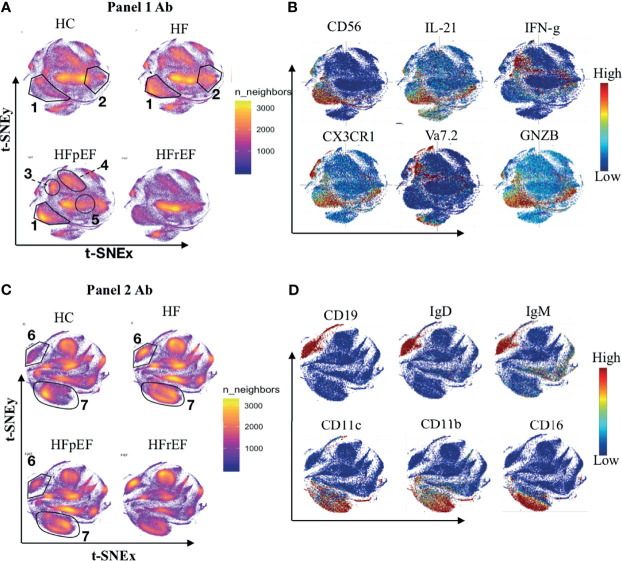
Distribution of immune cells in healthy controls patients with history of heart failure (HF). Heart disease was further categorized based on left ventricular ejection fraction levels into preserve ejection fraction (pEF) or reduced ejection fraction (rEF). **(A, C)** Figure shows distribution of immune cell types on t-SNE density map. Area with higher number of cells are reflected as dense clouds of yellow colors. For normalization, 100k cells were randomly sample from each category (HC n=18, CHF n=32, HFpEF n=14, HFrEF n=14) for visualization. **(B, D)** Expression of markers were overplayed on t-SNE map to show the phenotype of immune cells. **(A, B)** Shows the results from Panel 1 antibody staining while **(C, D)** shows results from Panel 2 antibody staining.

After acquisition, we performed dimensionality reduction using a t- distributed neighborhood embedding (t-SNE) algorithm. t-SNE summarizes the high-dimensional CyTOF data onto a 2-dimensional t-SNE map. The algorithm captures the expression patterns of all the markers and projects the cells with similar protein expression patterns closer on the 2D t-SNE map and places dissimilar cells further apart. t-SNE enabled dimensionality reduction and visualizations showed major differences in NK cell distribution in HF patients compared to HC (region 1 **Figure 1A**). The density of NK cells as highlighted in region 1 (**Figure 1A**) was higher in patients with HF (HF) compared to healthy controls (HC). The CD8+ T cell subset population was also perturbed and increased in HF cases (region 2, **Figure 1A**). Of relevance, the expanded NK cell population in patients from the HF group expressed CX3CR1, granzyme B, and IL-21(**Figure 1B**). Concurrently, the modulated CD8+ T cells expressed the pro-inflammatory cytokines TNF-α and IFN-γ (**Figure S1**). Data from panel 2 clearly showed a higher frequency of B cell cluster (region 6, **Figure 1C**) and overall higher frequency of monocyte/dendritic cells (region 7, **Figure 1C**) among PBMC from HF patients. Expanded B cell clusters expressed IgD and IgM (**Figure 1D**) suggesting an expansion of immature B cells in the peripheral blood of HF patients. Monocyte subsets (region 7, **Figure 1C**) expressed CD38 indicating increased inflammation in HF. These monocyte subsets also express pro-inflammatory cytokines IL-8 and IFN-γ (**Figure S2**).

To further understand the pathological immune mechanisms within two prominent subgroups of HF categorized based on left ventricular ejection fraction, we compared the immunome of HFpEF with that of HFrEF and found that the distribution of immune cells in HFpEF was distinct from that in HFrEF (**Figure 1A**). T-cell receptor (TCR) Vα7.2+ MAIT cells (region 3, **Figure 1A**), CD4+ T cell subsets (region 4, **Figure 1A**), and NK cells (region1, **Figure 1A**) showed higher density in HFpEF while B cell (Region 6, **Figure 1B**) and CD8+ T cell subsets (region 5, **Figure 1A**) had lower cell density in HFpEF compared to HFrEF. The expanded MAIT cells in HFpEF produced TNF-α and IFN-γ (**Figure S1**) suggesting an increased inflammatory activation in HFpEF.

### Innate and Innate-Like Cell Subsets Are Expanded and Inherently Pro-Inflammatory in HF

The t-SNE analysis showed modulation in major Immune cell subsets including NK, T cells, B cells, and monocytes in the HF patient cohort when compared with healthy subjects (**Figure 1**). To investigate the immune cell subsets at higher resolution, and to find the specific subsets dis-regulated within the major lineages, clustering and statistical testing were performed. K-means clustering was performed to group the immune cell subsets with a similar phenotype. The clustering algorithm enabled the unbiased grouping of cells and allowed the discovery of immune cell subsets with unique protein expression patterns. Immune cells were clustered into 80 nodes (subsets), the number of nodes were determined using the elbow method. The sums of variations at the various number of clusters were plotted to determine the elbow or inflection point where the phenotypic intra-nodal variations are balanced with the inter-nodal variations to estimate the number of nodes (clusters) that will provide an optimal number of unique yet internally phenotypic uniform cell population (**Figure S3**).

**Figure 2:**
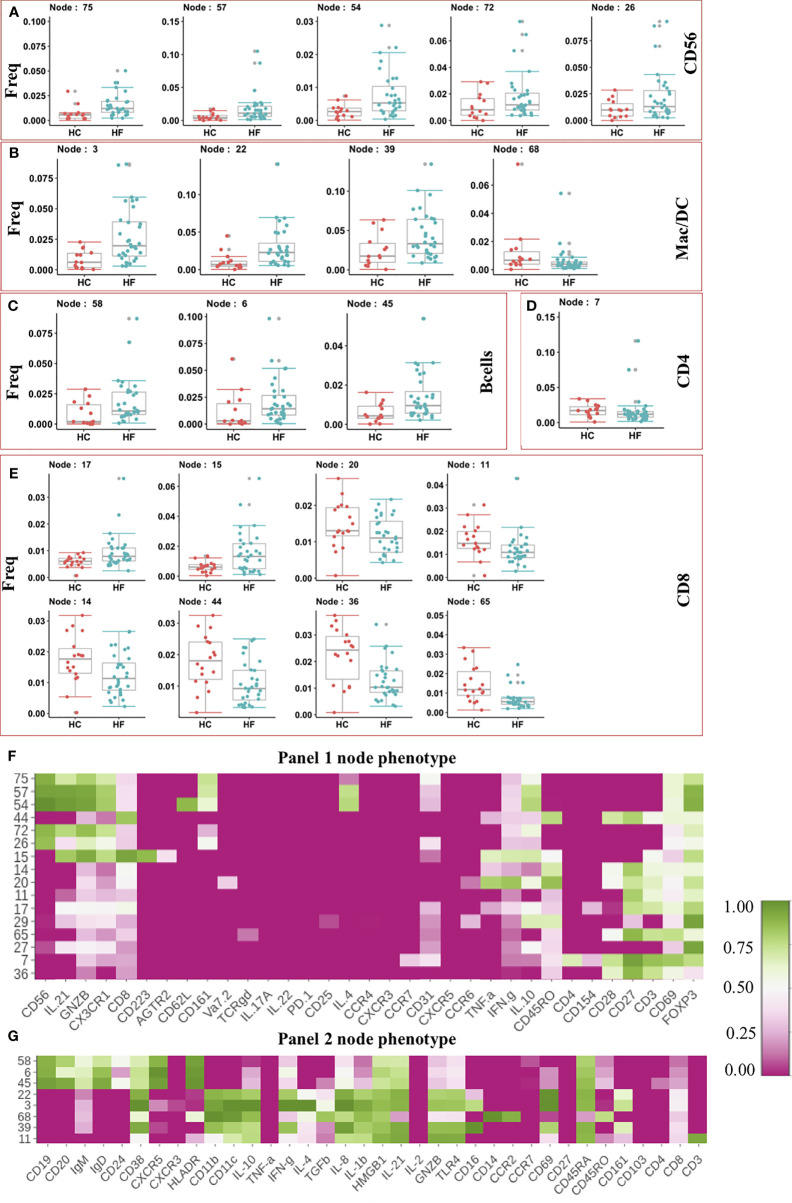
**(A–E)** Frequency of modulated Immune cell subset in Heart Failure (HF n=32) patients compared to Healthy Controls (HC, n=18). Frequency of significantly altered cell subsets in heart failure (HF) cases when compared to healthy control (HC) were shown as box and whisker plots. Phenotypes of the immune cell subsets that are significantly altered in Heart Failure cases are shown as heatmap. Median intensity was scaled normalized for heatmap visualization. Heatmap shows phenotypes of nodes found significantly altered from Panel 1 CyTOF analysis **(F)** and Panel 2 CyTOF analysis **(G)**.

After clustering, statistical testing was performed to find the significantly dysregulated subsets. Statistical analysis showed 5 NK cell nodes that were significantly (pval < 0.05) expanded in peripheral blood from HF patients compared to HC subjects (**Figure 2A**). These expanded NK cells nodes produced Granzyme B (GNZB) and expressed the Fractalkine receptor (CX3CR1) (**Figure 2F**). The Fractalkine (CXCL1) induces the transmigration of NK cells and induces the production of cytotoxic granules like GNZB and perforins. Fractalkine is expressed by endothelium during inflammation and helps the transmigration of CX3CR1 receptor-expressing NK cells into vascular tissue (24, 25). IL-21 enhances the proliferation and activation of NK cells (24). The expanded NK cells were also positive for IL-21 expression. Surprisingly two of these expanded NK cell nodes (Node 57, Node 54) also express anti-inflammatory cytokine IL-10 and IL-4 (**Figure 2F**). This could be a failed counterbalancing effort to the inflammation. Among the innate immune cell compartment (**Figure 2B**), 3 nodes (Node - 3, 22, 39) were significantly (pval < 0.05) expanded in HF while Node 68 showed lower frequency in HF compared to HC. The three expanded nodes showed phenotype (CD14-CD16+CD11c+IFN-γ+) of conventional dendritic cell (cDC) while node 68 showed the phenotype of classical monocyte (CD14+CD16-CCR2+) (**Figure 2G**). Expansion of pro-inflammatory cDC and contraction of classical monocyte nodes imbalances toward pro-inflammatory milieu in HF patients.

Together the expansion of cytotoxic NK cells and pro-inflammatory cDC suggests an increase in the immune-based inflammatory activation of innate lymphoid and myeloid cells in HF patients.

Interestingly, the adaptive arm of the Immunome in HF appeared to be less perturbed, and we did not observed expansion in most of the effector/inflammatory T cell subsets. The t-SNE analysis showed an increased frequency of B cell subsets in HF patients compared to HC (**Figure 1C**). Statistical testing further confirmed the t-SNE results showing 3 B cell nodes (Node 58,6,45) significantly expanded in HF patients (**Figure 2C**). These nodes showed similar phenotypes and expressed CXCR5, IgD, IgM, and HLA-DR (**Figure 2G**), indicating an activated functional phenotype. Within the T helper compartment, only a single CD4 T cell node (Node 7) was significantly reduced in HF compared to HC (**Figure 2D**). This subset (Node7) expressed CD69 and CD28 activation markers, however, production of typical cytokines like TNF-α or IFN-γ was not observed (**Figure 2E**), indicating a lack of inflammatory function or a potentially exhausted phenotype. Within the CD8 T cell compartment, two nodes (Node 15, Node 17) were expanded in HF while 6 nodes (Node 20, 11,14,44,36,65) had reduced frequency in HF patients’ blood (**Figure 2E**). Node 15 expressed CD223 (LAG3) inhibitory receptor. It also expressed cytotoxic marker enzyme GNZB along with TNF-α and IFN-γ production (**Figure 2F**) indicating its cytotoxic pro-inflammatory function (26). Node 20 showed IFN-γ, TNF-α, and CD45RO expression indicating a reduction in the pro-inflammatory memory CD8 T cell subset, while other significantly reduced CD8 T cell subsets (Nodes 11, 14, 17, 44, 36 and 65) did not show expression for any evaluated cytokines, cytotoxic or inhibitory molecule (**Figure 2F**). Overall, we did not observe major immune imbalance in the CD4 T cell compartment, while within the CD8 T cell compartment most subsets were significantly down-modulated in HF.

### From Immunome to Interactome: Increased Inter-Cellular Interactions of B and NK Cells in the HF Immunome Network

**Figure 3:**
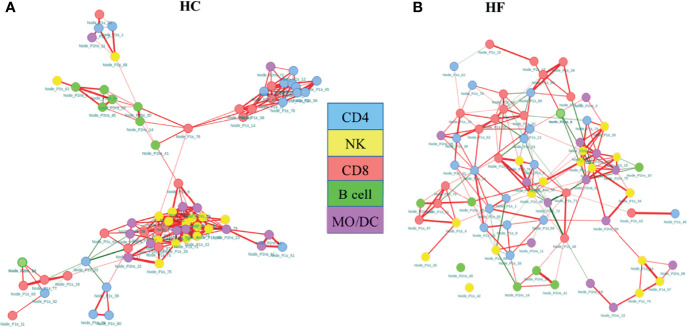
Co-regulatory network of immune subsets in heart failure (HF) disease shows highly modular structure compared to healthy controls (HC). Network analysis shows increased intracellular interaction of B and NK cells in the HF immunome network **(A)** compared to HC immune cell network **(B)**. Network of nodes (cellular subsets) were created and plotted as force directed layout. The color of the nodes indicates the cellular phenotypes. The edges (line connecting the two nodes) represent the connection between the nodes. The colors of the edges indicate the correlation direction: red = positive, green = negative. Absolute correlation cutoff of 0.6 or greater were used to established edge between two nodes.

Our statistical analysis clearly demonstrated immune dysregulation in HF patients compared to HC. However, to further investigate the immunome holistically at systems levels, we created an immune cell network to model inter- (interaction of subsets between the major lineage) and intra-cellular (interaction of subsets within the major lineage) interaction and evaluated properties of the overall immune response using a network analysis framework as described by *Kumar et al.* (27). Frequencies of T and NK cell nodes from Panel 1 staining and B and Mono/DC nodes from Panel 2 staining data were combined and pairwise correlation between each node was calculated. The strong correlations between the nodes were modeled as an interaction between the cellular subset. Based on correlations between the cellular subsets, correlation networks of HC and HF were created (**Figure 3**) as described in methods. Immune cell subsets were shown as node and interaction between the subsets as edges in the network. Network properties were calculated to understand the immunome of the HF compared to HC. The immune cell network of healthy subjects (**Figure 3A**) was strikingly different compared to that of HF patients (**Figure 3B**). Compared to the HF network (**Figure 3B**), the HC network (**Figure 3A**) showed a highly modular structure where major lineage subsets (Nodes) correlated very strongly among themselves and clustered together in the network. HC network modularity score (0.257) was higher than HF network modularity score (0.197). Specifically, B cells and NK cells nodes were grouped together in the HC network while in the HF network, B and NK cell nodes were scattered through the network and interacted more with subsets of other lineages. Node degree centrality in HF and HC network (**Figure S4**) further showed “rewiring” of the immunome. Degree centrality distribution of nodes showed larger changes in NK cell nodes. The Average degree centrality of NK cell nodes reduced to 5.88 in the HF network from 12.7 in the HC network (**Table S4**). The average degree centrality of NK cell nodes was higher in HC. However, most NK cell nodes edges were related among themselves (intra-lineage). In contrast, the average degree centrality of NK nodes was lower in HF but with more inter-lineage interactions, indicating a broader dispersion of interactions. Unlike NK cell nodes, B cell nodes degree centrality reduced marginally from 4.62 in HC to 4 in HF, however with an increase in inter-lineage interaction in HF compared with HC. The increase in B and NK cell interactions with other cell types possibly indicate their increased influence in modulating the immunome of HF patients. The betweenness centralization of the network was lower for the HF network (0.196) compared to the HC network (0.498) further corroborating the increase in inter-lineage interaction in the HF network. A higher centralization score in HC indicates that the network is controlled and influenced by fewer nodes and the overall network is organized around these central nodes.

The total number of edges, intended as interactions among the subsets, and edge density in HC was higher compared to HF (**Table 3**). Edge density is total number of edges divided by the total possible number of edges in the network and is a normalized value that could be compared across the network. Stronger intra-lineage interaction in HC results in higher edge number and edge density. Although the total number of edges was lower, the number of negatively correlated edges increased in HF compared to HC. The percentage of negatively correlated edges in HF was 11.8% while in HC it was only 2.1%. Together, network structure, modularity, centralization score, and edge density show greater interaction and potentially increased influence of NK and B cells in the immunome of HF patients.

**Table 3:** Properties of network.

property	HC	HF
**edge_density**	0.111894	0.062989
**centralization_betweenness**	0.498006	0.196534
**transitivity**	0.629032	0.53125
**modularity**	0.256749	0.199954
**average_path_length**	3.85446	4.871275
**no_neg_edges**	6	19
**no_pos_edges**	280	142

### The HFpEF Immunome Is Skewed Toward Inflammation

**Figure 4:**
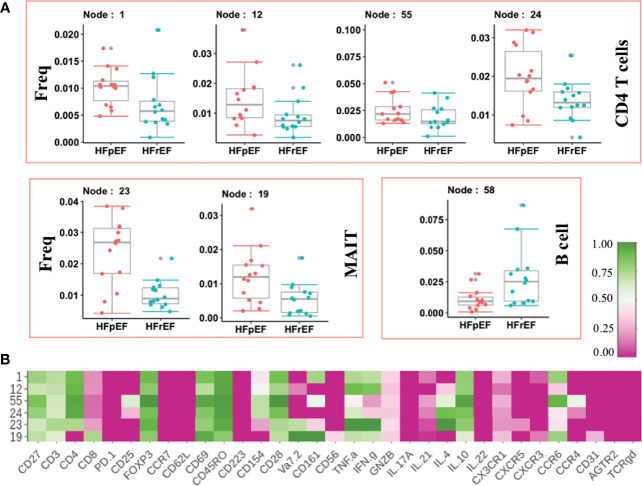
Immune cells modulated in Heart Failure cases categorized on the basis of left ventricular ejection fraction. **(A)** Frequency of significantly modulated Immune cell subset in patients with Heart Failure with preserved ejection fraction [HFpEF(n=14)] compared to Heart Failure patients with reduced ejection fraction [HFrEF(n=14)]. Frequency of significantly altered cell subsets is shown as box and whisker plots. **(B)** Phenotypes of the significantly altered immune subsets is shown as heatmap. Normalize median intensity of each marker used to plot heatmap.

HFpEF has distinct pathology compared to HFrEF as indicated by lack of response in HFpEF to therapies which offer benefit in HFrEF. We sought to identify the underlying systemic immune abnormalities in HFpEF that are distinct from HFrEF. We discovered significant expansion of inflammatory cytokines-producing CD4 T (Node 1,12, 24) and MAIT cell (Vα7.2+) subsets (Node 23, 19) (**Figure 4A**). Both MAIT cell and CD4 T cell subsets expressed TNF-α as well as IFN-γ (**Figure 4B**). Furthermore, expanded inflammatory CD4 T cell and MAIT cells expressed memory (CD45RO) and activation (CD69) markers, indicating chronic inflammation. A Treg subset (Node 55: CD4+CD25+FOXP3+) showed higher frequency in HFpEF. An increase in regulatory T cell frequency could be a failed response to an increase in inflammation. Of note, this expanded Treg node (Node 55) expressed CCR6, which inhibits Treg cell’s suppressive functions (28) thus probably explaining their inefficiency in controlling inflammation.

## Discussion

The accumulating evidence for the role of inflammation and immune mechanism in HF has led to clinical trials targeting inflammatory cytokines. Early trials showed no significant clinical benefit whereas the more recent CANTOS trial (20) of canakinumab show some promise. Reasons for varied results with anti-cytokine therapy are likely to be multifaceted, such as heterogeneous etiology, co-morbidities, the complex interplay between immune cells, and lack of knowledge on the different causes of inflammation. A systems immunology analysis approach could enhance the development of precision medicine for HF. To address the knowledge gap, we studied the Immunome of patients with HF and compared it with age-matched healthy subjects. Systems immunology led analysis of immunome showed systemic immune dysregulation favoring pro-inflammatory conditions in HF patients. Our study provides a rich resource and comprehensive insight into altered immune mechanisms in human chronic HF.

Human studies and animal models of acute myocardial injury have established a relationship between the onset of inflammation and the development of LV dysfunction and LV remodeling (11, 13, 29, 30). However, the role of the immune system in chronic HF and LV dysfunction is unclear. Nevertheless, despite limitations, various studies have highlighted links between inflammation and HF. However, these studies have focused on few specific predefined immune markers. This approach has the limitations typical of a Type 1 statistical error, where the entire experimental approach is focused on addressing a single, and often narrow, preconceived hypothesis. Instead, in our opinion, the investigational approach should be focused on a systemic and relational understanding of the systemic Immunome. Deep immuno-phenotyping using CyTOF technology, and our EPIC platform revealed modulation in all major immune cell subsets in HF compared to HC (**Figure 1**). A recent study (31) in pressure overload transverse aortic constriction (TAC) mouse model has used unbiased single cell sequencing approach to show that heart is infiltrated by all major cell subsets and many of them are dysregulated in the disease. Results from both the experimental HF model and our data from the human HF patients clearly suggests dysregulation in overall immunome rather than a specific immune cell subset.

We identified expansion of NK (CD56+CX3CR1+IL21+GNZB+) cells in patients with HF compared to HC subjects. We found no significant difference in any of the NK cell subsets in patients with HFpEF compared to patients with HFrEF. Contrary to our results, a clinical study by *Vredevoe* et al. (32) had shown reduction in NK cells in patients with HF. The *Vredevoe* et al. study only included patients with HFrEF and excluded patients with coronary artery disease. Moreover, we analyzed cells at higher resolution and found that only a few subsets were significantly expanded while many NK cell subsets were unaffected, demonstrating the advantage of unbiased deep immuno-phenotyping using CyTOF. NK cells limit viral replication, reduce inflammation in myocarditis, prevent collagen formation by cardiac fibroblast, and also play a protective role in myocarditis, and against cardiac fibrosis (33). However, NK cells are a double-edged weapon and could aggravate the inflammation by directly interacting with activating T cells for pro-inflammatory cytokine production. Expansion of NK cell subsets may potentially increase the inflammatory response in chronic HF. The previous studies have shown expansion in activated CD4 and CD8 T cells in TAC operated HF animal models (31, 34-36). The studies on T cell knock-outs and transgenic (36–38) OVA specific TAC mouse model have demonstrated a clear role of CD4 T cells in heart failure (36, 37) and showed CD8 T cells does not lead to heart failure even though higher CD8 T cells were present in myocardium of OVA antigen expressing transgenic mice after TAC (38). More recent studies showed depletion of CD8 T cells enhances early cardioprotective hypertrophy induced by pressure overload in TAC mouse model (35), prevents adverse ventricular remodeling, and improves cardiac function in acute myocardial infraction mice (39). However, we did not observe an increase in activated CD4 T cells from human HF patients (**Figure 2D**). Among CD8 T cell subsets, the majority of them showed lower frequency in human HF patients (**Figure 2E**). These differences in human disease and experimental disease models could be because of heterogeneity in human patients or may be specific to human or animal models and tissue analyzed. These differences in findings further necessitate the data from human studies for translation potential. The validation cycle from experimental model to clinical data will enhance the efficacy and safety during the drug discovery process.

Increased pro-inflammatory cytokines in peripheral blood from HF patients have been observed (8, 12). Our approach enabled the identification of the cell populations source of these cytokines. Indeed, we discovered an increased frequency of IL-1β producing cDC subsets in HF compared to HC. These expanded cDC subsets produced pro-inflammatory cytokine IFN-γ and expressed CD38 protein on the cell surface. CD38 expression is induced after activation in inflammatory conditions and regulates cytokine release and trafficking towards the site of inflammation (40). A recent single cell study (41) did not find any significant change in frequency of circulating monocytes in heart failure patients, however they reported increase in IL-1β in monocytes from HF cohort. An increase in these inflammatory myeloid cells reinforces heightened systemic inflammation in HF. A recent clinical trial (CANTOS) targeted IL-1β and showed reduced HF-related hospitalization in patients with previous myocardial infarction (21). Increase in IL-1β+ inflammatory M1-like macrophage is also reported in TAC mice further suggesting its role in heart failure and cardiac remodeling (31). We believe that specifically targeting the inflammatory cell subsets may further improve the treatment outcome in HF hospitalization and mortality. An increase in B1 (CD19+IgM+IgD+) cell subsets frequency in the circulation in HF further indicates chronic systemic inflammation corroborating the role of B cells in heart failure and possibly cardiac remodeling. IgM producing B1 cells lack specificity and recognize cell wall surfaces polysaccharides of pathogens (42). Overall, we found that in HF the architecture of the Immunome is profoundly perturbed, with a dominance of pro-inflammatory innate and innate-like immune cells. Importantly, to understand how various immune cell subsets interlace functionally together in a single network, correlation-based immune cell network was created. Methods and approaches from the social network analysis domain were used to understand the properties of the network at the system level. Strikingly, the immune cell network of HF patients was highly modular and dense compared to HC immune cell network. Change in modular structure in HF immune cell network indicates emergence of modules geared toward specific functions or potential loss of inter-cellular regulatory communication. In the HC network, NK and B cells were clustered together while in HF they were more scattered and showed increased interaction between subsets of another lineage type. B cells were abundant in normal mouse heart however, activated B cell subsets were enriched further in TAC disease animals (31). Our human data showed expansion in inflammatory B1 cells. Furthermore, our novel network analysis approach suggests increase in intercellular interaction of B cells with other immune cell subsets in HF patients that further corroborate the greater role of B cells in HF and cardiac remodeling. Statistical and Network analysis approach are complementary and can be used to priorities key potential mechanistic therapeutic targets. It’s the overall complex interplay and interactions among various subsets that finally results in disease phenotype. Network analysis framework helps to infer this cellular communication. Both in B and NK cells compartment, only subsets of cells were statistically significantly modulated in HF, however network analysis suggests greater changes in inter-cellular communication in other subsets further linking B cells and NK cells role in HF.

Within the HF subcategories, patients with HFpEF have been less studied, creating a knowledge gap in understanding the pathophysiology of this HF phenotype. We revealed systemic immune response dysregulation in HFpEF compared to HFrEF. Specifically, an increased frequency of pro-inflammatory CD4+ T and MAIT cell subsets in HFpEF compared to HFrEF. CD4+T and MAIT cell subsets produce TNF-α and IFN-γ. Our results suggest a dysregulated systemic immune response could be a major contributing causative mechanism in HFpEF and could provide an opportunity to discover novel biomarkers and therapeutic targets to stratify and treat such patients. Previous efforts to treat heart failure with anti -TNF therapeutics have shown mixed outcome in animal and mouse model. Various clinical trials did not find any clinical benefit, rather they report some adverse event (9, 18, 19). These trails mostly included patient with HFrEF. However, recent studies in human HFpEF and animal model have shown benefit with dapagliflozin and dapagliflozin (43, 44). These drugs are sodium-glucose cotransporter 2 inhibitor; however, they report inhibition of markedly upregulated TNF-α in human HFpEF and in pig model of HFpEF. These studies again emphasize the utility of targeting inflammatory cytokines to treat heart failure. These existing drugs could be explored further to understand the mechanism of inflammatory cytokine suppression without adverse event for better and more effective HFpEF therapeutic development.

Small cohort size prevented the immunome analysis in comorbidities stratified HF subgroups and is a limitation of the study. Despite large heterogeneity within chronic HF, we discovered dysregulated immune cell subsets in HF, that could be further pursued for interventional study to benefit broader group of HF patients.

In summary, we employed high dimensional CyTOF technology to investigate the immunome of HF patients and discovered immune pro-inflammatory immune dysregulation. Particularly, pro-inflammatory innate lymphoid and myeloid cells were expanded and showed altered intercellular communication in the immune cell network of HF compared to the immune cell network of healthy controls. Furthermore, we identified inflammatory subsets expanded specifically in the HFpEF category providing biomarker and therapeutic targets for specifically targeting inflammation-mediated HF pathology in this difficult and increasingly prevalent form of HF.

## Data Availability Statement

The original contributions presented in the study are included in the article/**Supplementary Material**. Raw mass cytometry data files are deposited to FlowRepository (Repository Id: FR-FCM-Z4VU). Further inquiries can be directed to the corresponding authors.

## Ethics Statement

The studies involving human participants were reviewed and approved by NUHS, NHCS and KKH Central Institutional Review Board (CIRB). The patients/participants provided their written informed consent to participate in this study.

## Author Contributions

PK performed the experiments and bioinformatics analysis. AL, SP, NB, CC, and SH performed the experiments. TK and VS recruited the patients obtained the relevant blood samples. TA and JY recruited healthy control subjects. AR, TA, JY, and CL participated in study design, grant acquisition, and patient recruitment and manuscript preparation. PK, AR, and SA lead the study and wrote the manuscript. AR and SA conceived the study and arranged funding for the study. All authors contributed to the article and approved the submitted version.

## Funding

This study was supported by grants from the NMRC (NMRC/MOHIAFCAT2/005/2015, NMRC/TCR/0015-NCC/2016, NMRC/OFLCG/002/2018, MH 095:003\016-0002, MH 095:003\016-0001, CIRG19may0052, NMRC/TA/0059/2017), Duke-NUS and SingHealth AMC core funding. This research was also supported by the National Research Foundation Singapore under its NMRC Centre Grant Program (NMRC/CG/M003/2017) and administered by the Singapore Ministry of Health’s National Medical Research Council. The ATTRaCT study was supported by research grants from A*STAR Biomedical Research Council ATTRaCT program [SPF2014/003, SPF2014/004, SPF2014/005]. The funder had no role in study design, data collection and analysis, decision to publish, or preparation of the manuscript.

## Conflict of Interest

The authors declare that the research was conducted in the absence of any commercial or financial relationships that could be construed as a potential conflict of interest.

## Publisher’s Note

All claims expressed in this article are solely those of the authors and do not necessarily represent those of their affiliated organizations, or those of the publisher, the editors and the reviewers. Any product that may be evaluated in this article, or claim that may be made by its manufacturer, is not guaranteed or endorsed by the publisher.
