# Supplementary material for: Pro-Inflammatory Derangement of the Immuno-Interactome in Heart Failure
Source: Front Immunol. 2022 Mar 15;13:817514. doi: 10.3389/fimmu.2022.817514 (PMC8964981; doi:10.3389/fimmu.2022.817514)
Supplement: Supplementary file 1 [file DataSheet_1.pdf]

Supplementary Material

Supplementary Tables:

Table S1: Clinical Information and demographics of human subjects

Table S2: Panel 1 Antibodies conjugated metal, clone and vendor information

Table S3: Panel 2 Antibodies conjugated metal, clone and vendor information

Table S4: Mean degree centrality of major immune cell subsets in HC and HF network

Supplementary Figures:

Figure S1: Marker expression overlaid on t-SNE map (Panel 1)
Expression of indicated markers were overlaid on t-SNE map to shows the cellular phenotype ont-SNE map

Figure S2: Marker expression overlaid on t-SNE map (Panel 2)
Expression of indicated markers were overlaid on t-SNE map to shows the cellular phenotype ont-SNE map

Figure S3: Elbow plot
Within group of sum of square was plotted on y axis for increasing number of clusters on x-axis

Figure S4: Degree Centrality of immune cell subsets
Node degree centrality of HC and HF immunome network was plotted as bar graph. Color of graph shows the major cell lineage type

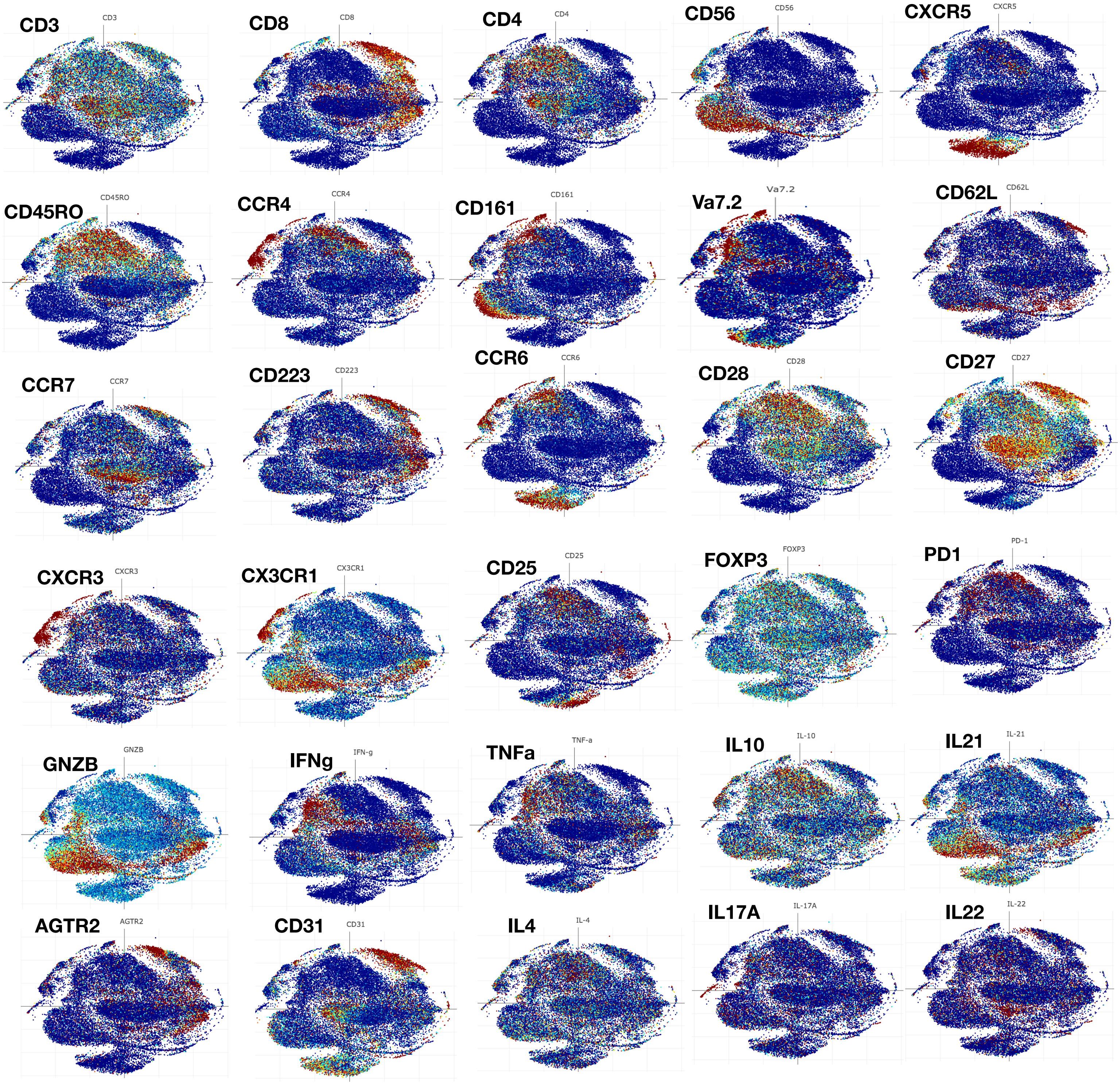

Figure S1: Marker expression overlaid on t-SNE map (Panel 1)

Expression of indicated markers were overlaid on t-SNE map to shows the cellular phenotype ont-SNE map

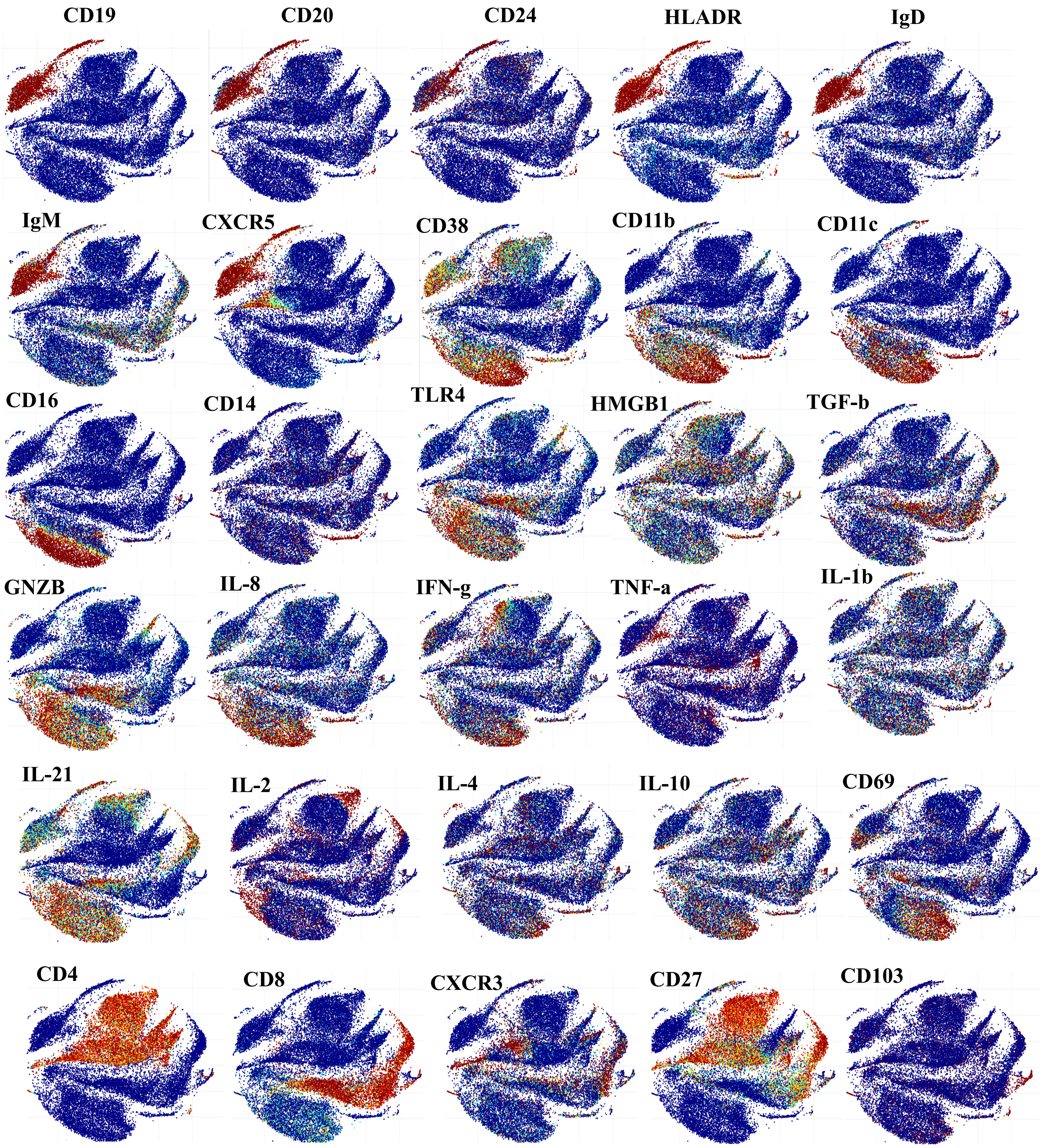

Figure S2: Marker expression overlaid on t-SNE map (Panel 2)

Expression of indicated markers were overlaid on t-SNE map to shows the cellular phenotype ont-SNE map

A **Panel 1**

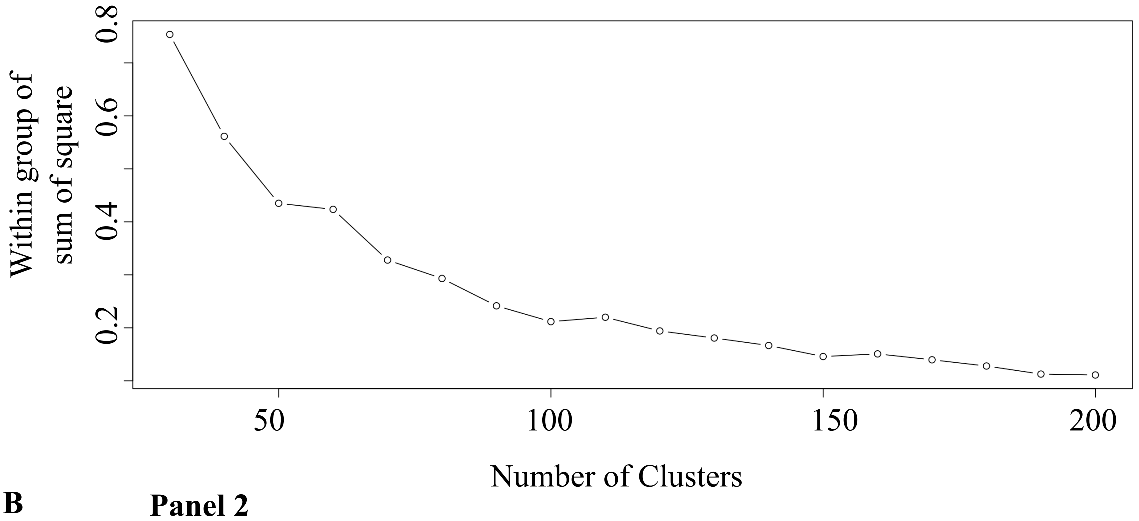

B **Panel 2**

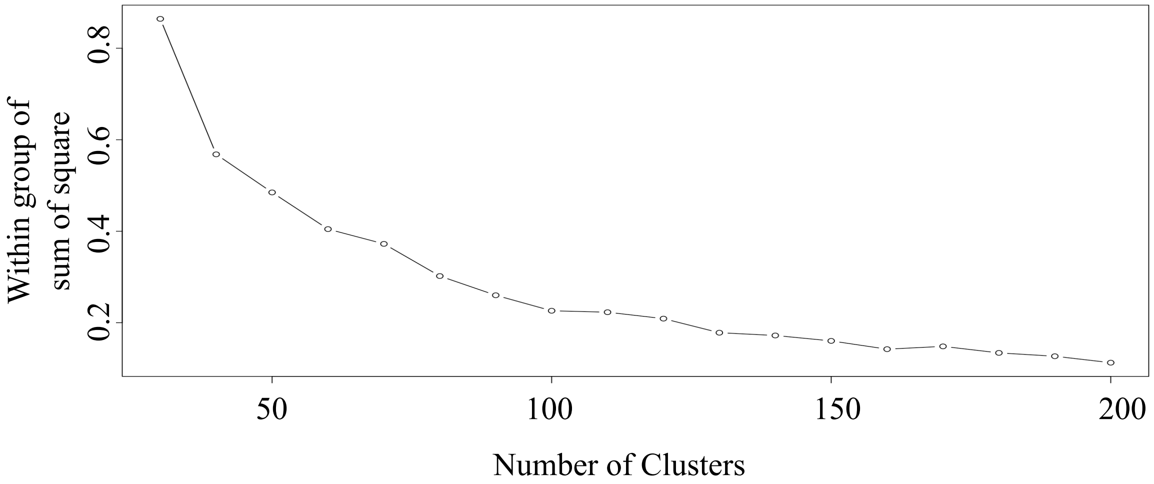

Figure S3: Elbow plot

Within group of sum of square was plotted on y axis for increasing number of clusters on x-axis

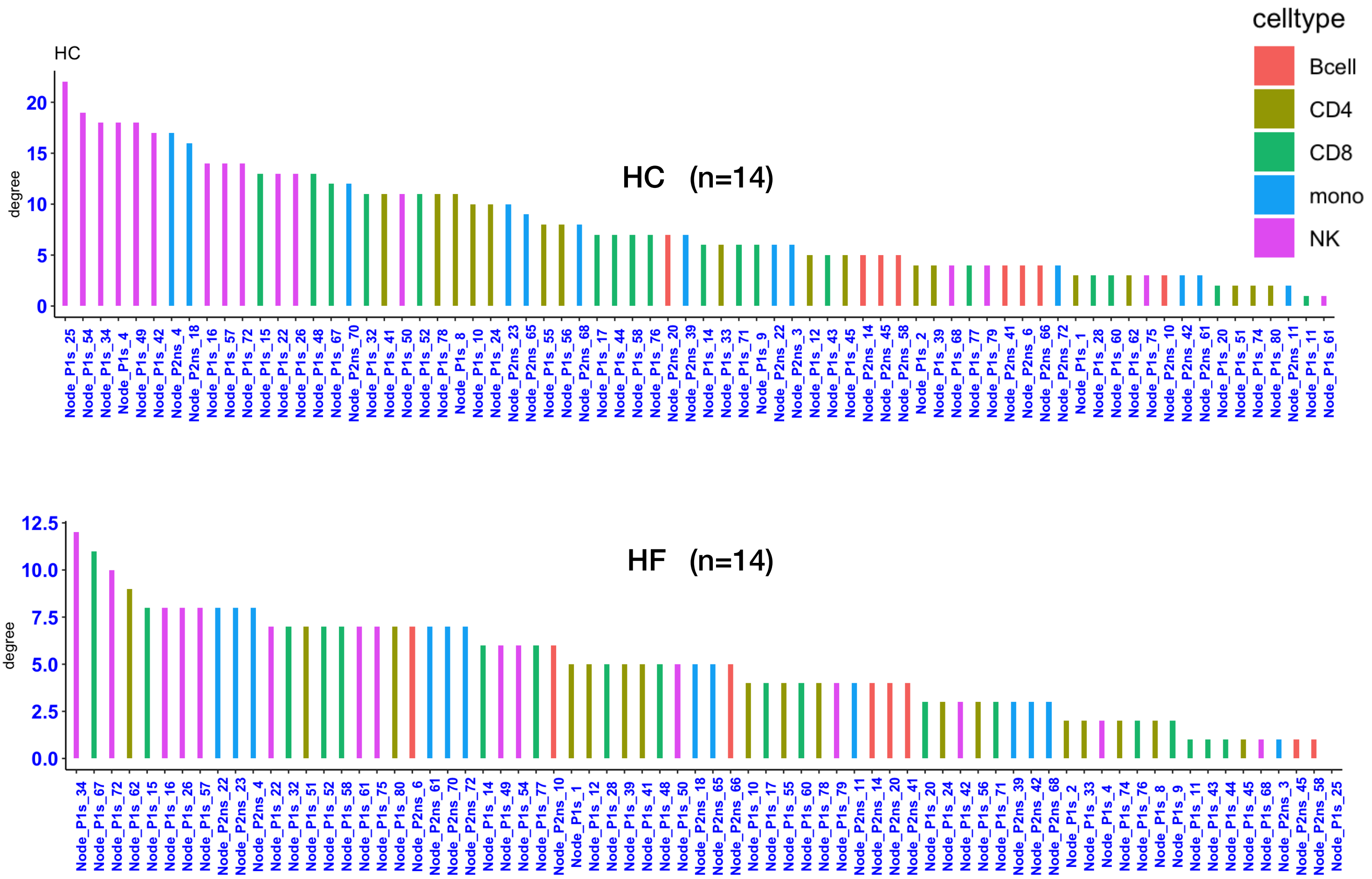

Figure S4: Degree Centrality of immune cell subsets
Node degree centrality of HC and HF immunome network was plotted as bar graph.
Color of graph shows the major cell lineage type
